# Paving the physician-scientist career path: from grassroots gathering to national forum

**DOI:** 10.1172/jci.insight.192689

**Published:** 2025-04-08

**Authors:** Kyu Y. Rhee, Charles W. Emala, Emily Jane Gallagher, Don C. Rockey, Patrick J. Hu, Jatin M. Vyas, Daniel P. Cook, Tiffany C. Scharschmidt, Olujimi A. Ajijola, ASCI Research Pathways Working Group, Christopher S. Williams

**Affiliations:** 1 Division of Infectious Diseases, Department of Medicine, and; 2 Department of Microbiology and Immunology, Weill Cornell Medicine, New York, New York, USA.; 3 Department of Anesthesiology, Columbia University, Vagelos College of Physicians and Surgeons, New York, New York, USA.; 4 Division of Endocrinology, Diabetes and Bone Disease, Department of Medicine, Icahn School of Medicine at Mount Sinai, New York, New York, USA.; 5 Digestive Disease Research Center, Medical University of South Carolina, Charleston, South Carolina, USA.; 6 Department of Medicine, Vanderbilt University School of Medicine, Nashville, Tennessee, USA.; 7 Division of Infectious Disease, Department of Medicine, Columbia University, Vagelos College of Physicians and Surgeons, New York, New York, USA.; 8 Division of Pulmonary, Critical Care, and Occupational Medicine, Department of Medicine, University of Iowa, Iowa City, Iowa, USA.; 9 Department of Dermatology, UCSF, San Francisco, California, USA.; 10 UCLA Cardiac Arrhythmia Center and UCLA-Caltech Medical Scientist Training Program, Los Angeles, California, USA.; 11 The ASCI Research Pathways Working Group is detailed in Supplemental Acknowledgments.; 12 Program in Cancer Biology, Vanderbilt University School of Medicine, Nashville, Tennessee, USA.; 13 Veterans Affairs Tennessee Valley Healthcare System, Nashville, Tennessee, USA.; 14 Vanderbilt Ingram Cancer Center, Vanderbilt University School of Medicine, Nashville, Tennessee, USA.

## Abstract

The Alliance for Academic Internal Medicine (AAIM) first convened a workshop in 2015 that brought a small group of internal medicine program directors together who recognized the growing success of early-phase physician-scientist training programs but the unclear path afterward for these trainees. The meeting subsequently evolved into what is now the annual American Society for Clinical Investigation/AAIM/Burroughs Wellcome Fund (ASCI/AAIM/BWF) Physician-Scientist Pathways Workshop, which continues to bring stakeholders together to discuss the obstacles to success that physician-scientists face at all stages of their careers. This perspective presents the history and goals of the workshop, with an emphasis on the most recent meeting in 2024, and looks ahead to the work that still needs to be done to ensure a robust physician-scientist workforce.

## Introduction

Few contributions more clearly distinguish the societal importance of academic medicine than those of the physician-scientist. Physician-scientists are often viewed as the crown jewels of many academic medical centers due to their unique ability to integrate their training in clinical medicine and the scientific method to directly identify and solve problems of clearly defined clinical relevance in a focused and intentional manner. As such, many academic medical centers have invested heavily in establishing and maintaining a pipeline to recruit and train future generations of physician-scientists. To date, such investments have paid handsome dividends, as reflected by the integration of research into many medical school curricula and the evolving and expanding effect of MD-PhD training programs on the biomedical research workforce. However, despite the extent of such investments and their short-term success, the career path for physician-scientists beyond medical school remains surprisingly compartmentalized, fragmented, and underdeveloped.

In 2015, the Alliance for Academic Internal Medicine (AAIM) convened a workshop that recognized the growing success of early-phase physician-scientist training programs (PSTP) but the incompletely developed nature of the downstream career path. This workshop sought to identify issues pertinent not only to the postgraduate training phase but also its integration with pregraduate training programs and, ultimately, the transition to full professional independence. A central recommendation that emerged from this meeting was the establishment of an annual workshop specifically for program directors involved in research training. The annual (and recently renamed) American Society for Clinical Investigation/AAIM/Burroughs Wellcome Fund (ASCI/AAIM/BWF) Physician-Scientist Pathways Workshop has since become an annual cornerstone gathering for stakeholders across the spectrum of physician-scientist training, with the shared goal of creating a more structured and integrated career pathway for individuals to pursue the physician-scientist career path in an intentional and achievable, rather than purely aspirational, manner. Below, we provide a brief historical overview and summary of key goals and milestones in its development. We then outline plans to further pave and formalize the career path for physician-scientists, with the hope of enlisting the full spectrum of stakeholders invested in their unique and specific ability to advance human health.

## Genesis: the 2015 AAIM convocation and birth of a workshop

The 2015 AAIM summit brought a small group of internal medicine program directors together and acted as a catalyst for the Research-in-Residency/PSTP (RiR/PSTP) Directors Workshop. Topics of initial discussion centered on broad foundational issues, such as identifying the scope and goals of RiR and PSTP programs as essential components of attracting and supporting physician-scientists; developing effective mentorship strategies for research trainees as a crucial element of career support; securing funding for research training positions as a fundamental requirement for sustaining the workforce; navigating the complexities of integrating research training within residency curricula to ensure adequate research time and training; and advocating for the pivotal role of physician-scientists and the physician-scientist training pipeline in biomedical research.

This initial gathering, perhaps most significantly, established a formal platform for dialogue and collaboration among program directors committed to tackling the obstacles to physician-scientist career development.

## Growth and recognition: scaling up

Following its inception, the workshop grew in size and influence, reflecting the increased recognition of the importance of physician-scientist training as a national priority. Several additional key milestones were achieved during this period.

### Broader stakeholder engagement.

The workshop attracted a more diverse audience, including program directors from specialties other than internal medicine, representatives from the NIH and other funding agencies, the American Board of Internal Medicine, and even trainees themselves.

### Collaboration with key stakeholders.

The workshop began to forge ties with other organizations committed to supporting and developing physician-scientists, such as the ASCI and the BWF, leading to enhanced visibility and commitments to sustained support.

### Dissemination of knowledge.

Fostering an environment of open discourse and intellectual exchanges, the workshop participants importantly collaborated to share the concepts discussed with the broader community, by publishing manuscripts related to physician-scientist training (Table 1).

## The ASCI/AAIM/BWF partnership and a new venue: a new era

In 2022, the workshop began to be held in conjunction with the Association of American Physicians (AAP)/ASCI/American Physician Scientists Association (APSA) Joint Meeting, with critical support from the ASCI, in addition to AAIM. This intentional move placed the workshop on the Sunday following the conclusion of the main meeting, a strategic effort that created synergy with the Joint Meeting. Furthermore, the BWF became an important partner, providing both scholarly and financial support during this time frame, which helped to further elevate this conference. This synergy added the following elements.

### Enhanced programming.

The workshop now features cutting-edge research presentations, career development components, and dedicated sessions on diversity, equity, and inclusion in physician-scientist training, all benefiting from the broader scientific focus of the joint meeting.

### Expanded stakeholder attendance.

Senior faculty, chairs, vice chairs, and deans with interests in physician-scientist training attending the Joint Meeting found value in attending, contributing to and learning concepts from the workshop to bring back to their home institutions.

### Further integration with trainees and junior faculty.

Conference programming and participation now includes input from the ASCI PSTP Emerging Generation Awardees (E-gen) and Young Physician-Scientist Awardees (YPSA), which provides valuable input and leadership development from trainees and faculty during these early career stages.

### Increased national impact.

The workshop has become a key platform for disseminating best practices, influencing national policy, and advocating for sustained funding for physician-scientist training, amplified by the larger audience attending the joint meeting.

### Broader representation across clinical medicine.

The workshop now involves presenters and attendees across a broad range of clinical specialties, expanding the effect of best practices in physician-scientist development beyond internal medicine.

### A shared vision.

The ASCI/AAIM/BWF partnership has solidified a shared commitment to nurturing the next generation of physician-scientists and ensuring their continued success in academic medicine and beyond.

### Synergy with APSA.

Critically, the workshop is held the same morning as the APSA-sponsored Residency Luncheon, a popular recruiting event for trainees attending the national meeting and PSTP directors. The proximity of these 2 events has created a natural flow of attendees between both functions, substantially increasing attendance at both the workshop and the luncheon and fostering more significant interaction between program directors and trainees.

## A catalyst for scholarly output

As alluded to above, the workshops have also served as a nidus for scholarly work in the field of physician-scientist training. The collaborative discussions, breakout sessions, and shared experiences among attendees have led to several important publications addressing critical issues in the field (Table 1). These publications, often arising from consensus generated at the workshops, have significantly advanced the national discourse on physician-scientist training and provided valuable guidance for program directors, trainees, and institutions.

## Highlights from the 2024 ASCI/AAIM/BWF workshop (April 7, 2024, Chicago, Illinois, USA)

**Figure 1:**
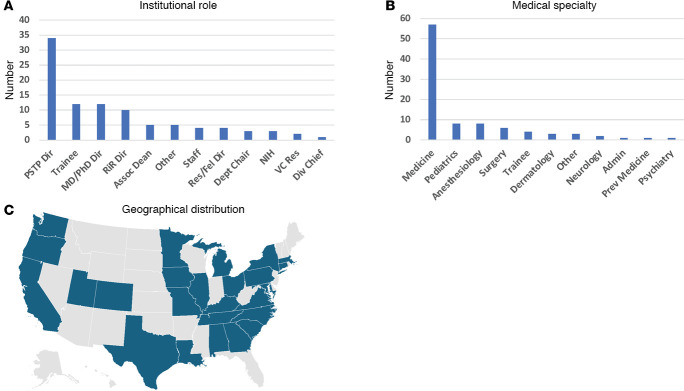
Attendee demographic data from the 2024 Research Pathway Directors Workshop. (**A**) Institutional role of workshop participants. (**B**) Medical specialty of workshop participants. (**C**) Geographic distribution of those participating in the workshop. Res., residents; Fel.; fellows; VC Res., vice chair for research.

The 2024 ASCI/AAIM/BWF workshop was entitled “Reimagining Support & Pathways for Physician-Scientists: A Collaborative Approach to Enhancing Diversity, Mentorship, and Infrastructure” and featured a broad geographic representation, and more than 100 participants from various specialties and institutional roles (Figure 1) established a direct dialogue with the NIH and major philanthropic funding organizations centered around support for physician-scientists. This dialog included a keynote address from Ericka Boone, Director of the NIH Division of Biomedical Research Workforce; a panel discussion among program officers from 5 different NIH institutes (NCI, NHLBI, NIDDK, NIAID, NIGMS), the VA health system, and 4 philanthropic foundations that specifically support physician-scientist career development awards (CDAs; BWF, American Heart Association [AHA], Damon Runyon Cancer Foundation, and the Physician-Scientist Support Foundation [PSSF]); 3 breakout discussion sessions focused on: (a) departmental and institutional funding challenges for early-stage physician-scientists; (b) optimizing the timelines for transitioning to independence; and (c) defining the value proposition of physician-scientist training; showcasing three quick-shot presentations of recent literature on physician-scientist training. Key takeaways from each component are summarized below (Table 2).

### Working together to support the future of the biomedical research workforce: 

### recent NIH initiatives

 The keynote presentation noted that continued growth of the biomedical research workforce faces 3 major challenges: (a) the increased length of time for education and training and increased age at first faculty appointment, resulting in low pay and subsequent effects on the mental health and retention of biomedical researchers; (b) the paucity of postdocs and insufficient role models, particularly among women and underrepresented individuals, at each academic rank; (c) growing rates of imposter syndrome, harassment, isolation, and aggressions in the workplace.

Funding is also viewed as a potential barrier, as traditional physician-scientist K awards, K08 for basic research and K23 for clinical research, have seen only modest and variable increases in salary support across NIH institutes. The K99/R00 mechanism (1) — which seeks to support the transition of outstanding postdoctoral researchers with a research and/or clinical doctorate degree from mentored, postdoctoral research positions to independent, tenure-track or equivalent faculty positions and is offered by many NIH institutes — is not often utilized by physician-scientists, despite dedicated awards for physician-scientists offered by the NIAlD and NIDCR. Efforts have been put forward to address help in promoting the physician-scientist career path. Five NIH institutes (NHLBI, NIAID, NCI, NIA, NEI) have established R38/K38 programs to stimulate research during residency. The NIH Loan Repayment Program (LRP), which was similarly established to support and retain physician-scientists in the academic community and biomedical research, provides up to $50,000 per year in loan repayment assistance in 2-year contracts (renewable biennially) and has a 50% success rate. The Medical Scientist Training Program (MSTP), which was originally established in 1964 to address the chronic shortage of physician-scientists engaged in biomedical research, continues to be viewed by NIH as a success (2).

A 2021 analysis of the National Research Service Award (NRSA) fellowship program review process emphasized the following: (a) specific focus on 3 key elements of applications, including potential of the applicant, strength of the science, and quality of training plan; (b) defining criteria to give less advantaged applicants a better chance of success without disadvantaging others; and (c) reducing bias in review by preventing inappropriate consideration of sponsor and institutional reputation.

The NIH has also developed programs, policies, and tools focused on supporting early stage and diversity in the biomedical workforce. Two supplements; the Research Continuity Supplement, an award to enhance the retention of investigators facing critical life events and the Re-Entry and Re-Integration Supplement for individuals seeking to reenter an active research career after an interruption for family responsibilities or other qualifying circumstances were highlighted (3). Eleven NIH institutes participate in the Research Opportunities for New and “At-Risk” Investigators to Promote Workforce Diversity (4).

### The role of the NIH, VA, and foundations in support of physician-scientists: 

### CDA panel discussion

This session focused on the many nuances and differences among CDAs, even among K mechanisms across different NIH institutes, including differences in eligibility criteria, award duration, and allowable salary support. The importance of various components of the K application is often variable and dependent on the specific NIH institute, with some institutes weighing achievement more heavily than potential, weighing the research plan more heavily than training and mentorship plans, and vice versa. Additional institutional data and recommendations can found in Supplemental Tables 1–3 and Supplemental Figures 1 and 2 (supplemental material available online with this article; https://doi.org/10.1172/jci.insight.192689DS1). It is essential that applicants pay careful attention to the stated goals of each CDA program. Many K applications are not selected for funding upon first submission, so it is essential to view the necessity to resubmit not as a failure but as a demonstration of resilience in biomedical research.

### Critical issues for physician-scientists: breakout sessions

#### Supporting the salary gap between physician-scientists and clinicians.

The gap between a T32 stipend and a postgraduate year (PGY) or junior faculty salary is frequently substantial, particularly for those in procedural subspecialties, and is often an unrecognized financial challenge for academic medical centers. K awards from most NIH institutes pose a similar challenge as they do not provide 75% of a clinical salary, despite the requirement of 75% protected research time. Therefore, institutions/departments still need to supplement the salary of junior faculty on K awards. While the NIH allows certain procedure-based specialties to decrease the protected research time to 50% so clinical practice can help supplement physician-scientist salaries, it remains to be determined whether this allowance to reduce the protected research time should be expanded to other clinical specialties, to help institutions support physician-scientists, or if this reduction in protected research time and greater clinical commitment ultimately hinders the future physician-scientist’s career. A fundamental, yet unresolved, topic is whether a physician-scientist salary should approximate that of a full-time clinician salary in the same field and, if so, how.

#### Optimizing the timelines for transitioning to independence.

The time to what could be considered “success” as an independent researcher by traditional metrics (e.g., time to first R01) is generally agreed to be too long (5). Potential solutions included encouraging physician-scientists to apply earlier for K awards, applying for K99/R00 mechanisms, applying earlier for R01s, or perhaps skipping the K mechanism entirely and applying directly for R01s; however, such approaches would require significant changes on the part of institutional training programs and NIH grant review mechanisms. Questions about how to support new physician-scientists without individual extramural funding also arose, in particular whether such individuals should be appointed into instructor positions that do not start the academic tenure clock or assistant professor positions that often command higher salaries.

#### Defining the value proposition of physician-scientists.

Institutions vary in their philosophical support of physician-scientists. As of 2024, business models and compensation paradigms in many academic medical centers have shifted squarely toward focusing on clinical revenues. Physician-scientist–derived innovation enhances the reputation of academic medical centers (through NIH and other funding) and branding but lacks quantification. Many institutions consider the cost of recruiting someone at the assistant professor level to be substantially greater than training and retaining a trainee to join their own faculty. Physician-scientists bring publicly funded knowledge and skills obtained from their extensive training, to benefit for-profit companies without compensating academic medical centers or funding agencies, raising the possibility of developing collaborative funding models between industry, government, and academic institutions.

### Recent highlights from the physician-scientist literature (quick shots summary)

Gallagher et al. questioned whether it is time to reduce the length of time of postgraduate training for physician-scientists in internal medicine. This study identified a median time of 8.3 years for MD-PhDs to achieve a CDA (e.g., NIH K award) with those with MD-only credentials requiring a median of 9.4 years and recommended approaches to shorten this time requirement to enhance retention of this critical biomedical work force (5). Jansen et al. identified a lack of financial and childcare support for physician-scientist trainees with parenting responsibilities. These deficiencies were seen as an impediment to sustaining the physician-scientist workforce and as challenges to creating diversity in this workforce — a workforce that often requires a decade of training during traditional ages of child-rearing. The NIH and individual academic institutions were challenged to address these issues with financial support of childcare and family-related costs that reflect the true cost of living, flexibility in grant timelines, and a livable stipend (6). Emala et al. addressed the chronic dearth of physician-scientist trainees who enter several clinical specialties, including anesthesiology. The study compared the specialty choices of research-oriented medical students entering 11 medical specialties over a recent decade and addresses the diversity of those trainees entering anesthesiology. Several recommendations were put forth to enhance the recruitment, retention, and research support, primarily from anesthesiology foundations, for aspiring physician-scientists within anesthesiology (7).

## Future-forward: the ASCI/AAIM/BWF Physician-Scientist Pathways Workshop in 2025 and beyond

Looking ahead, we ask what’s next? An overarching goal of the ASCI/AAIM/BWF Physician-Scientist Pathways Workshop series is to promote collaboration, mentorship, and innovation to create an infrastructure that can facilitate the success and longevity of physician-scientist careers. Therefore, each workshop will concretely focus on actionable strategies, drawing from institutional best practices, mentorship models, and funding opportunities to advance physician-scientists’ careers. Each year, the program will address a critical theme relevant to all stages of the career path and, thus, provide value to all participants, from undergraduate recruitment to faculty development. Topics of current interest include the following: (a) strategies to increase the number and diversity of individuals entering the career path across the entire spectrum of training; (b) innovations in training that will increase the accessibility and effect of the career path, including historically marginalized communities and clinicians working in underserved areas; (c) strategies to support specific transitions across the career spectrum, including premedical to medical school, medical school to residency, clinical training back to research, trainee to junior faculty, and others; (d) strategies to sustain extramurally funded scientific productivity, balance clinical and research roles, and cultivate a culture of pragmatic mentorship that promotes excellence and inclusivity as a foundation for both the individual and broader physician-scientist community; and (e) strategies to engage institutional leadership and create a structured environment where institutions actively provide resources, mentorship, and development opportunities tailored specifically for physician-scientists.

Participants in the workshop can, thus, look forward to in-depth discussions on evolving trends, innovative practices, and emerging challenges shaping the future landscape for physician-scientists. However, to pave the entire length of the physician-scientist career path, it will be particularly incumbent on this workshop to focus its interests in the pedagogical, administrative, and financial aspects of the career path in a strategically actionable way. We, therefore, welcome all those on the path to join us.

## Author contributions

KYR, CWE, and EJG have been designated as co–first authors. This order was determined by the timing of their contributions.

## Supplementary Material

Supplemental data

## Figures and Tables

**Table 1:**
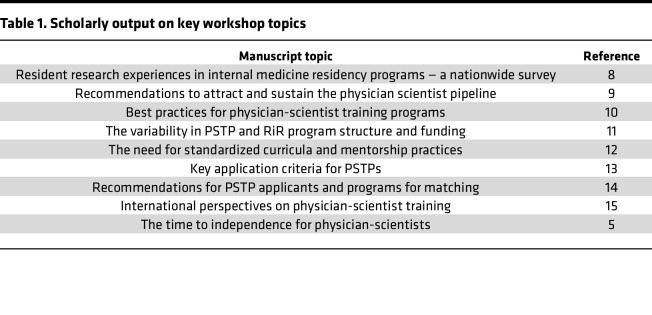
Scholarly output on key workshop topics

**Table 2:**
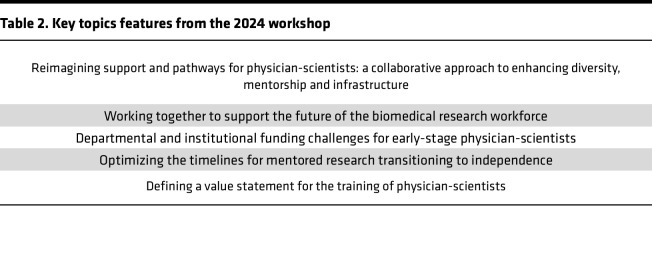
Key topics features from the 2024 workshop

## Version 1 04/08/2025

Electronic publication
