# Supplementary material for: Paving the physician-scientist career path: from grassroots gathering to national forum
Source: JCI Insight. 2025 Apr 8;10(7):e192689. doi: 10.1172/jci.insight.192689 (PMC11981613; doi:10.1172/jci.insight.192689)
Supplement: Supplemental data [file jciinsight-10-192689-s024.pdf]

Summary of Features of Career Development Awards for Physician-Scientists

ASCI/AAIM/BWF
Research Pathway Directors Annual Workshop

Sunday, April 7, 2024, 7:00 am-12 pm Central
Swissotel, Chicago

Theme: Reimagining Support & Pathways for Physician-Scientists:
A Collaborative Approach to Enhancing Diversity, Mentorship, and Infrastructure

Supplementary Information - Table of Contents

Page 3-4. Supplementary Table 1. NIH institutes supporting different K08 mechanisms

Page 4. NCI and ARHQ- specific K08 mechanisms

Page 5-6. Supplementary Table 2. NIH institutes supporting different K23 mechanisms

Page 7. Supplementary Table 3. Eligibility criteria based on career stage

Page 8. Supplementary Figure 1. Eligibility criteria based on total years of KL2 and K08/K23 support

Page 9. Other eligibility criteria for K08/ K23 awards

Page 10. Protected time

Page 11. Supplementary Figure 2. Salary and supply budget

Page 12. K99/R00 for Physician Scientists – NIAID

Page 13. Administrative supplements for critical life events

Page 14. VA Career Development Awards (IK2)

Page 15. 2024 Organizing Committee

Supplementary Table 1. K08

NIH Institute	PA-24-181 Clinical Trials Required	PA-24-182 Clinical Trials Not Allowed	PA-24-183 BESH	PAR-24-217 NIH HEAL PCTP Clinical Trials Required	PAR-24-218 NIH HEAL PCTP BESH	PAR-24-219. NIH HEAL PCTP Clinical Trials Not Allowed
NEI	✓	✓	✓	✗	✗	✗
NHLBI	✓	✓	✗	✗	✗	✗
NHGRI	✓	✓	✗	✗	✗	✗
NIA	✓	✓	✓	✓	✓	✓
NIAAA	✓	✓	✓	✓	✓	✓
NIAID	✗	✓	✗	✗	✗	✗
NIAMS	✗	✓	✗	✓	✓	✓
NIBIB	✓	✓	✗	✓	✗	✓
NICHD	✓	✓	✓	✓	✓	✓
NIDCD	✓	✓	✓	✗	✗	✗
NIDCR	✗	✓	✗	✗	✗	✓
NIDDK	✗	✓	✗	✓	✗	✗
NIDA	✓	✓	✓	✗	✗	✗
NIEHS	✓	✓	✓	✗	✗	✗
NIGMS	✗	✓	✗	✗	✗	✗
NIMH	✓	✓	✓	✗	✗	✗
NINDS	✓	✓	✓	✓	✓	✓

NIMHD	✓	✓	✗	✗	✗	✗
NLM	✓	✓	✗	✗	✗	✗
NCCIH	✓	✓	✓	✓	✓	✓
NCI	✓	✓	✓	✓	✓	✓
BESH: Basic Experimental Studies Involving Humans; HEAL: Helping End Addiction Longterm; PCTP: PainCare Cllinician Training Program						

Other NCI K08 Awards

PAR-24-319 Continuing Umbrella of Research Experiences (CURE) Clinical Trials Required

PAR-24-320 CURE Clinical Trials Not Allowed

AHRQ K08 Awards

PA-22-232: AHRQ Mentored Clinical Scientist Research Career Development Award

PA-22-050: AHRQ Patient-Centered Outcomes Research (PCOR) Mentored Clinical Scientist Career Development Award

Supplementary Table 2. K23

	PAR-24-220 NIH HEAL PCTP Clinical Trial Required	PA-24-186 BESH	PA-24-185 Clinical Trial not Allowed	PA-24-184 Clinical Trial Required	PAS-22-207 Substance Use Prevention and Treatment Clinical Trial Required
NEI	✗	✓	✓	✓	✗
NHLBI	✗	✗	✓	✓	✗
NHGRI	✗	✗	✗	✗	✗
NIA	✓	✓	✓	✓	✗
NIAAA	✓	✓	✓	✓	✓
NIAID	✗	✗	✓	✗	✗
NIAMS	✓	✗	✓	✓	✗
NIBIB	✓	✗	✓	✓	✗
NICHD	✓	✓	✓	✓	✗
NIDCD	✗	✓	✓	✓	✗
NIDCR	✗	✗	✓	✗	✗
NIDDK	✓	✗	✓	✓	✗
NIDA	✗	✓	✓	✓	✓
NIEHS	✗	✓	✓	✓	✗
NIGMS	✗	✗	✗	✓	✗
NIMH	✗	✓	✓	✓	✓
NINDS	✓	✓	✓	✓	✗
NIMHD	✗	✗	✓	✓	✗

NLM	✗	✗	✗	✗	✗
NCCIH	✓	✓	✓	✓	✓
NCI	✗	✗	✗	✗	✓
BESH: Basic Experimental Studies Involving Humans; HEAL: Helping End Addiction Longterm; PCTP: PainCare Cllinician Training Program					

Eligibility Criteria Based on Career Stage

Supplementary Table 3.

NIH Institute	Eligible
NIAAA	**At the time of award**, the candidate must have a secured **full-time faculty or faculty equivalent appointment** in an appropriate research-intensive environment
NIGMS	Eligible candidates … must have **completed postgraduate clinical training and have secured a faculty appointment** in an appropriate research-intensive environment at the time of the award
NIMH	**No more than 6 years** of postdoctoral research experience **at the time of application** initial or resubmission
NCI	Postdoctoral or non-tenured junior faculty Assistant Professor, not Associate Professor
NIDCR	Postdoctoral or early career faculty
NIEHS	Should have **completed clinical training** and should be board-eligible **by the time of award**
NIBIB	**Within 10 years** of completing residency/fellowship
NINDS	**Within 5 years** of completing clinical training

References:
https://grants.nih.gov/grants/guide/contacts/IC-specific-information-PA-24-181.html
https://grants.nih.gov/grants/guide/contacts/IC-specific-information-PA-24-182.html
https://grants.nih.gov/grants/guide/contacts/IC-specific-information-PA-24-183.html
https://grants.nih.gov/grants/guide/contacts/IC-specific-information-PA-24-184.html
https://grants.nih.gov/grants/guide/contacts/IC-specific-information-PA-24-185.html

Eligibility Criteria Based on Total Years of KL2 and K08/K23 Support

Supplementary Figure 1.

- **NCI:** No Limit
- **NHLBI:** 8 years
- **NINDS:** 8 years
- **NIDA:** 6 years and email NIDA prior to submitting
- **NEI:** 6 years
- **NICHD:** 6 years
- **NIEHS:** 6 years
- **AHRQ:** 5 years

Other Eligibility Criteria for K08/ K23 Awards

Need active US medical license

US Citizen, Non-citizen, or Permanent Residency Requirement
at time of award not when applying

Protected Time

75% Effort for Research & Career Development Activities*

***Exceptions: Minimum 50% Effort Allowed For Some Procedural Specialties**

***NOT-HL-22-022:** NHLBI: surgeons, interventional cardiologists, electrophysiology cardiologists, interventional pulmonologists, and interventional radiologists

***NOT-CA-21-054:** NCI: U.S.-licensed surgeon-scientists with active surgical duties

***NOT-DK-25-002:** NIDDK: All surgeon-scientists in sub-specialties recognized by the American College of Surgeons, as well as transplant surgeons

***NOT-MD-24-017:** NIMHD: Surgeons and clinicians in procedure-intensive specialties. a surgeon is a physician who is clinically active in one of the 14 surgical subspecialties recognized by the American College of Surgeons. Clinicians in procedure-intensive specialties include but are not limited to specialties such as: interventional cardiology, interventional radiology, gastroenterology, interventional radiology, etc.

*__NOT-AG-21-019:__ NIA: Surgeons who are clinically active in one of the 14 surgical subspecialties recognized by the American College of Surgeons

***NIAMS:** Orthopedic scientists https://www.niams.nih.gov/grants-funding/research-training-and-career-development-programs/research-career-development-awards

***NOT-HS-22-018:** AHRQ: Surgeons

NIDCD: Surgeon-scientists https://www.nidcd.nih.gov/training/career-development-awards-k-series#K08

NIMH: Neurosurgeons https://www.nimh.nih.gov/funding/training/table-of-k-parameters

NINDS: Neurosurgeons https://www.ninds.nih.gov/funding/training-career-development/clinician-scientist/mentored-clinical-scientist-research-career-development-award

Salary and Supply Budget

Supplementary Figure 2.

Institute(s)	Budget
NCCIH, NICHD, NIDCR, NIDDK, NIMHD, NHLBI*, NIA*	• Salary: $100,000<br>• Supplies: $25,000<br>• *Up to $50,000 for patient-oriented research
NIAMS	• Salary: $100,000<br>• Supplies: $30,000
NIAAA, NIAID, NIDA, NIDCD, NIGMS, NIMH	• Salary: $100,000<br>• Supplies: $50,000
NIBIB	• Salary: $105,000<br>• Supplies: $40,000

Institute(s)	Budget
NLM, NINDS*	• Salary: $125,000<br>• Supplies: $50,000<br>• * & 1000
NHGRI	• Salary: Commensurate with the applicant institution's salary structure for persons of equivalent qualifications, experience, and rank.<br>• Supplies: $40,000
NIEHS	• US Legislative Salary Cap<br>• Supplies: $40,000
NCI	• US Legislative Salary Cap<br>• Supplies: $50,000
NEI	• US Legislative Salary Cap<br>• Supplies: $25,000 up to $50,000 with sufficient justification

K99/R00 for Physician Scientists – NIAID

NIAID Physician-Scientist Pathway to Independence Award – Independent Clinical Trial Not Allowed

https://grants.nih.gov/grants/guide/pa-files/PAR-23-071.html

NIAID Physician-Scientist Pathway to Independence Award – Clinical Trial Required

https://grants.nih.gov/grants/guide/pa-files/PAR-23-070.html

Administrative Supplements for Critical Life Events
NOT-OD-23-031

This supplement program is intended to ensure continuity of research among recipients of mentored career development K awards by providing supplemental research support to help sustain the investigator’s research during a period in which the PD/PI experiences critical life events which have the potential to impact research progress or potential productivity.

For the purposes of this program, critical life events that would qualify for consideration include childbirth, adoption, serious personal health issues or illness and/or debilitating conditions, high-risk pregnancy, and primary caregiving responsibilities of an ailing spouse, child, partner, parent or a member of the immediate family during the project.

The administrative supplement budget is limited to 1 year. The application budget cannot exceed a maximum direct cost of $70,000

https://grants.nih.gov/grants/guide/notice-files/NOT-OD-23-031.html

VA Career Development Awards: CDA-2 (IK2)

Candidates for Career Development Awards need not have a VA appointment at the time they apply but must be nominated by a VA facility and must identify an appropriate VA mentor.

The sponsoring VA research office must submit the application on behalf of the nominee.

Funding duration: 3-5 years

Salary Support: Full Specialty Specific Salary Support

Research Support: $65,000/year

https://www.research.va.gov/funding/cdp.cfm

ASCI Research Pathways Working Group

Name	Affiliation
Ajijola, Olujimi	University of California, Los Angeles
Barnard, Kristin	Alliance for Academic Internal Medicine
Baron, Rebecca M.	Brigham and Women's Hospital, Harvard University
Burns, Audrea	Baylor College of Medicine
Choate, Keith	Yale University
Cooper, Paige	Burroughs Wellcome Fund
Eltzschig, Holger K	University of Texas Health Science Center at Houston
Gallagher, Emily	Icahn School of Medicine at Mount Sinai
Gruber, Peter	Yale University
Hawley, John	American Society for Clinical Investigation
Hu, Patrick J	Vanderbilt University Medical Center
Mosammaparast, Nima	Washington University in St. Louis
McGarry, Colleen	American Society for Clinical Investigation
Pittenger, Christopher	Yale University
Rhee, Kyu Y.	Weill Cornell Medicine
Rockey, Don C.	Medical University of South Carolina
Scharschmidt, Tiffany	University of California, San Francisco
Steed, Ashley	Washington University in St. Louis
Williams, Christopher S	Vanderbilt University Medical Center
